# Annotations

**Published:** 1889-07-20

**Authors:** 


### Annotations.

**Barmaids in Council.**

And why not? How many weeks is it ago that the "literary ladies" of the metropolis came together to dine and remained to smoke? What is sauce for the "bluestocking" may well be sauce for the "frizzed hair." The lady members of the "Bar" met together to the number of about seventy last week, and seem to have had a thoroughly good time of it. The happy thought of inviting those hard-worked and much-enduring young women to a *conversazione* seems to have originated with Lord and Lady Kinnaird, who accordingly met them at the Morley Rooms, Bedford-row, on Tuesday afternoon. These rooms, many readers will be glad to know, have been set apart for the exclusive use of barmaids by the Young Women's Christian Association, and many thousands of them have found there a welcome refuge, and much needed rest for mind and body. We are not surprised to be told that a sumptuous tea was provided. Young women who preside over the Bath buns and sandwiches, the pork pies and cold chicken of refreshment bars, could not be expected to sit down to anything "plain." It is not reported that they indulged in cigarettes; and we may well believe that at the Morley Rooms they certainly did not. They did, however, thoroughly enjoy themselves, if much laughter and many games be evidences of enjoyment. The young girl who elects to be a barmaid probably does so very often without knowing very well what she is about. She chooses an occupation in which it is very difficult indeed for her to retain any of the fresh bloom of girlhood. Nevertheless, to their honour be it said, very many barmaids preserve a simplicity and wholesomeness of life and manner which are truly surprising. Surrounded as they are by temptation and coarseness of all kinds, those who stand against deteriorating influences have their characters greatly strengthened, and are able to show a good example and to exert an invaluable influence on their less experienced sisters. Lord and Lady Kinnaird and their coadjutors of the Young Women's Christian Association never did a better work than when they stretched out hands of sympathy and encouragement to barmaids. We heartily congratulate both the givers and the sharers of last Tuesday's entertainment. Young girls who have to face the difficulties and dangers of the barmaid's career can have no safer and more trustworthy friends than the members of the Young Women's Christian Association.

**A New Brain Wanted.**

London is neither deaf nor blind, but it is afflicted with paralysis. Not, perhaps, the "general paralysis of the insane," but the peculiar kind of paralysis which one can imagine would result if an elephant had the brain and spinal cord of a mouse. Perhaps there is not a single grown up man in the metropolis or its suburbs who does not see with the utmost clearness, and understand in the thoroughest possible way, that the Thames, which in its natural condition was a fine river, has now become an open drain which gives off into the surrounding atmosphere all the concentrated stinks of the largest city in the world. The only reason why this is so, is the paralysis already indicated. We have elephantine size of limb, and we have clearness of eye, but there is no nerve centre of brain and spinal cord which can send sufficient energy into the large body to make it remove its sewage in the only safe and rational way. The voice of reason proclaims in the London wilderness that no solid sewage should on any condition be thrown either into the river or into the sea. The solid sewage should all be returned to the land. Though all the engineers and all the chemists in the world should say "No," they would all be wrong. Nature says "Yes." When you put sewage into the soil Nature smiles her approval by absorbing all its dangerous odours at once, and giving you back a splendid crop of corn, roots, or grass. When you put it anywhere else, either into the river or into the sea, she compels you to see how senseless is the course pursued, by killing the fish and sending forth odours most miserable to endure and most destructive to health and life. What more convincing facts than these are wanted? Have not chemists and engineers experimented for a sufficient length of time on our noble river? Where is the practical result? The present writer has crossed London Bridge twice a day for the last two months. He still survives, thanks to an agricultural constitution. But the stinks! Who shall describe them? Whilst we are wasting our solid sewage, and destroying both the river and ourselves in the process, the soil in Kent and the other home counties is crying out for more and better manure. In many places the land is being so exhausted that much of it is no longer worth cultivation. The writer spoke to a farmer the other day about some of his fields which are now lying as waste as a common. His reply was that they had had no manure put into them for twenty years, and that consequently no crop could grow which would pay for seed and labour. Chemists and engineers, we insist, are not competent to advise on the sewage question unless at the same time they are practical agriculturists. It is admitted that a new scheme for removing the solid sewage to the soil would be costly and troublesome. But whatever the cost, and whatever the trouble, it is the only rational thing to be done. Were it done, our river would be as beautiful and healthy as it is now hideous and dangerous, and the country round about London might be converted into one of the most fruitful regions in the world. Is the County Council the new brain that is to make the municipal elephant convert his potential strength into actual and successful work?

**Men or Women.**

It is said that at a recent meeting of the British Nurses' Association a proposal was made to appoint a committee of "men of business" to conduct the affairs of the association. This proposal was, however, negatived on the ground that to place the business of the association in the hands of such a committee would be, from the ladies' point of view, to "hand them over to their enemies." What is the meaning of this? It must be either that men and women are natural enemies, or that the majority of the members of the British Nurses' Association look upon men, or at least upon men of business, as their foes. The idea that there is a natural antagonism between men and women is too absurd for discussion; and if it be the fact that the British Nurses' Association has succeeded in turning men of business into "enemies," it can only be because that association has schemes to propose or objects to gain which do not commend themselves to the honest and candid intelligence of those best qualified to judge. We do not wish in any way to bear hardly upon the association. It is never very English to strike a man when he is down, much less a woman. And now that the association has been compelled to lower its flag, and to eat a little humble pie, we would be very considerate towards its better-disposed members. We cannot, however, forbear pointing out that not long ago much temper was displayed, and unfounded accusations were flung about recklessly in all directions. Those accusations, like wide-flying birds, have come home at last to roost, and now it is seen that motives imputed without justification, and suspicion engendered without ground, are resulting in the discrediting of the accusers themselves. The association will do well to seek counsel outside its own membership, even, perhaps, at the hands of men of business. We do not say that women cannot manage their own affairs; but we do say that men and women together are much more likely to arrive at just and reasonable conclusions than women alone. The British Nurses' Association started off on a wrong tack when it raised its voice against men. Let it admit its faults, come back to common sense, cease its railings, and generally behave as if it were animated by a little human kindness and sweet reasonableness, even towards men. Nothing is so offensive to right-minded persons as eternal shrewishness. It may be taken for piquant seasoning once or twice; but when it is continued for weeks or months, everybody learns to regard it as a nuisance or worse. If the association would but once learn that the way to win public favour is to render disinterested public service, it might even yet hope to survive and be of use to its members.

